# Developmental Trends in Mother-Infant Interaction from 4-Months to 42-Months: Using an Observation Technique

**DOI:** 10.2188/jea.JE20090176

**Published:** 2010-03-05

**Authors:** Masatoshi Kawai, Kumiko Namba, Yuko Yato, Koichi Negayama, Shunya Sogon, Hatsumi Yamamoto, Japan Children’s Study Group

**Affiliations:** 1 Japan Science and Technology Agency (JST)/Research Institute of Science and Technology for Society (RISTEX); 2 Center for the Study of Child Development, Institute for Education, Mukogawa Women’s University, Nishinomiya, Hyogo, Japan; 3 College of Letters, Ritsumeikan University, Kyoto, Japan; 4 Faculty of Human Sciences, Waseda University, Tokorozawa, Saitama, Japan; 5 Department of Human Relations, Kyoto Koka Women’s University, Kyoto, Japan; 6 Clinical Research Institute, Mie-chuo Medical Center, National Hospital Organization, Tsu, Mie, Japan

**Keywords:** longitudinal study, mother-infant interaction, observation method, joint attention

## Abstract

**Background:**

It is clear that early social interaction follows from mother-infant interaction after pregnancy. Many researchers have illuminated this interaction in the first years of life. Most common mother-infant interaction is the *attachment behavior* of an infant. The Japan Children’s Study (JCS) development psychology group hypothesis is that the early mother-infant interaction will predict later social behaviors. But the method applied to evaluate this interaction mainly comes from the evaluation of the whole observation situation and is dependent upon the *coder*. We applied a new observational method that checked the on/off status of behavior and recorded sequentially.

**Methods:**

Using a semi-structured observation setting as our method, we analyzed the developmental change of mother-infant interaction within a *toy situation*.

**Results:**

The result indicated that mother-infant interaction with a toy altered at around 9-months and is salient to the usual developmental change of *joint attention*. Additionally *cluster analysis* suggested that the developmental pattern is divided into two clusters. This is the first report on a developmental pattern of joint attention.

**Conclusions:**

These results indicated that the developmental trend of gaze direction and vocalization is one candidate of measure for evaluating the mother infant social interaction from the point of joint attention.

## BACKGROUND

In the last two decades, social ability vulnerabilities are becoming increasingly evident in our society and in schools. These problems range from AD/HD, Asperger’s Syndrome to daily communications such as classroom interaction from elementary school age. As many studies were conducted to identify the mechanism of this problem, we still do not know much about the root causes.

The JCS research group aims for a clear understanding of the mechanism of this problem. We set the *sociability in school* as a main outcome, and analyze the relationship between early sociability exposure such as infant medical condition, infant psychological factors and the environmental condition. The missions of Developmental Psychology Group in JCS are: 1. To develop a template for developmental testing that will measure the same aspects/traits across all infants. 2. To develop an evaluation situation for early mother-infant interaction as a precursor for studying later social interaction.

For the purpose of developmental evaluation, we choose the *KIDS* (Kinder Infant Development Scale)^1^ test. This test includes the following six features; physical ability, verbal ability, cognitive abilities, social behavior for adults, social behavior for children, and manipulation. The results derived from this test are both DQ and pass/fail items for behaviors such as “Baby can walk by his/her self ” etc.

Observation and recording of mother-infant interaction was the core process of this study. In this particular investigation, the study cohort was exceptionally large, it was difficult to use direct observation because of the problem of validity and reliability. Evaluating the behavior through observer’s eyes required cross-referencing for research reliability. It is clear that a well-trained rater can evaluate the mother-infant interaction using a set coding description. However, for collecting data from multiple locations and at multiple times as in this large quantitative research project, it would have generated unreliable qualitative results due to the difficulties in maintaining consistency in observation/evaluation recording and analysis. In this study, we applied a microanalysis model for evaluating mother-infant interaction.

The purpose of this report is to demonstrate the method which applied to this large cohort study in analyzing the mother-infant interaction from 4 to 42 months of age.

### Applying the observation for cohort study and method for analysis

Evaluating early mother-infant interaction is important in predicting social development. The purpose of JCS is to identify the relationships between sociability in elementary school pupils as an outcome of their former pre-schooling social development exposure.

The relations of early mother-infant interaction and later social development have been discussed.^2^^,^^3^ In the field of mother-infant interaction study, the method for collection the interaction data is mainly by the observation and evaluation by observers. For example, “Strange Situation” developed by Mary Ainsworth and her associates^4^ is a commonly used procedure for assessing individual differences in attachment.^3^ These procedures require for training for identifying the various categories. For applying this in research situations, formal training and standard reliability testing are essential, but is difficult to conduct with a very large number of subjects. Regardless of Goldberg’s relatively positive comment on this method,^5^ we did not use this scenario for cohort studies due to cost-effectiveness issues. In a longitudinal study, it is difficult to keep the evaluation level sustained for such a long period of time and across a wide spread area.

It is obvious that spontaneous mother-infant interaction without any control is less stressful and more likely to effect a more natural and valid set of conditions for collecting data on the behaviors of mother and infant.

To resolve this issue, in 2006 JCS decided to record the observation situation and conduct analysis after observation. Observation time (approx. 40 minutes) included observations by pediatricians and cognitive psychology groups, with developmental psychology group observation time usually limited to 10 minutes.

The purpose of this article is to discover the developmental trends of mother-infant interaction by using the observation method.

### Observations of the mother-infant interaction

#### Data acquisition

Mother and infant were video recorded in a 3.72 m × 3.72 m laboratory playroom equipped with four remotely controlled cameras. This playroom can be arranged in a variety of ways allowing freedom of infant movement. Age 4, 9, 18, 30 and 42 month infants with their mothers were encouraged to interact for 2 to 3 minutes in an infant seat. The conditions provided different kinds of opportunities for mother to interact with their babies through visual, auditory and proprioceptive modalities. Toys were available to use in mother-infant interaction. We used the same set of toys for appropriate age in each observation sites of JCS. Instructions to the mothers were “play and talk to the baby as you would normally do at home”.

#### Data reduction

Due to the continuous-time process analysis goal of JCS, and an attempt to consider a wide variety of both mothers and infants from interaction observations, data reduction procedures are more complex than in most studies. In this section the general approach is outlined. The database is a transcription, coded continuously in time of the actions of both infant and mother, using a set of coding categories (Table 1). The times and codes were entered into a computer database. Coders on this phase of the analysis are blind to the subjects’ scores on other tests. The database combined and sorted categories, incorporating the recorded data for finer analysis, and rapidly derived sequential statistics. We are no longer enslaved by costly months of hand derivation, nor are bound by the original set of coding categories. In this paper, we derived the 5 second time sampling data of behavior category form original time continuous data set for identifying the early mother-infant communication in a free play situation.

**Table 1.** An example of coding category using JCS

**Infant**							
Facial expression	1.positive	2.negative	3.frown	4.neutral	5.other		
Vocal	1.on	2.off					
Body	1.front	2.right	3.left	4.lean forward	5.bend back	6.other	
Arm	1.lower hand	2.reaching	3.other				
Hand touch	1.mouth	2.face	3.body	4.cloth	5.other hand	6.other	
Leg	1.normal	2.right	3.left	4.front	5.back	6.other	
Head	1.normal	2.right	3.left	4.front	5.back	6.other	7.other
Gaze at	1.mother	2.self	3.right	4.left	5.up	6.down	

**Mother**							
Facial expression	1.positive	2.negative	3.frown	4.neutral	5.other		
Vocal	1.on	2.off					
Touch	1.face	2.hand/arm	3.foot	4.body	5.other		
Gaze	1.baby	2.self	3.right	4.left	5.up	6.down	7.other
Body	1.upright	2.lean	3.back	4.right	5.left	6.other	
Getting attention	1.point	2.showing	3.give	4.demo	5.physi-ori	6.other	

Coders are trained to reach a minimum reliability of a 0.85 proportion of agreement (Cohen’s Kappa).^6^ For research reliability, a well-trained second coder, working independently, coded at least 5 cases of mother-infant interaction. These “reliability coders” conducted discussions at points of disagreement and lead to refinements in behavioral coding. An agreement is scored if a coder records the onset of the same category within three seconds of that scored by the reliability coder. Random checking operated at least at 3 month intervals for one or two rounds of recording, with discussions occurring if disagreement/queries arose.

#### Data management for cohort study

**Figure 1.**
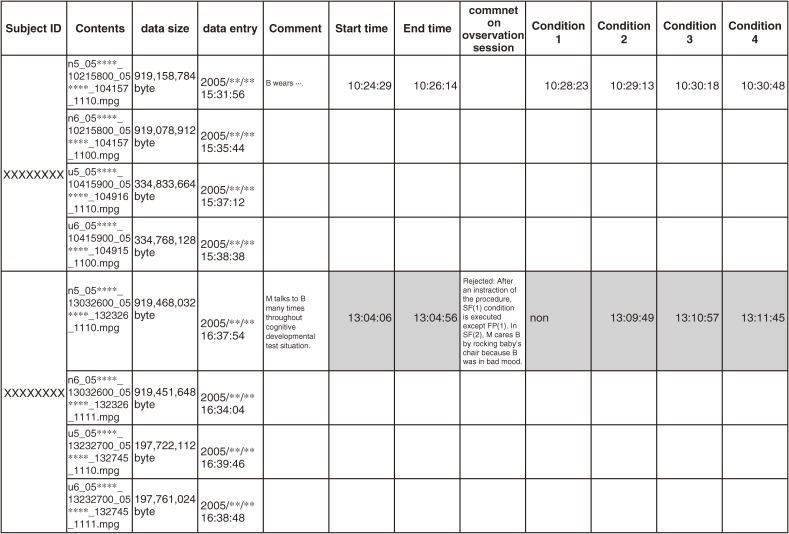
Recording book and contents.  A sample of the record-book for two subjects. Because of the file size limit, observation data are automatically divided into 3 files as in second row for one subject. A line appears at the top of table to indicate information about observation session (Subject ID, related 3File name, data size, date of processing) and the information about continuous observation session includes the subject's condition and the start time for Free Interaction and Still Face conditions. Shaded cell sections were not for analysis. These data tables are projected for use for both data coding and future evaluation of mother-infant interaction.

For applying this method to this cohort, we prepared the record book for each subject from first observation to last. This book including the ID, area where the data was collected, date of observation, any event and time occurred during observation, file name, size, coder’s name, the time access for updating the data and the name of the file handler (Figure 1).

### Developmental change of mother infant interaction from 4 to 42 month using by observation method

**Figure 2.**
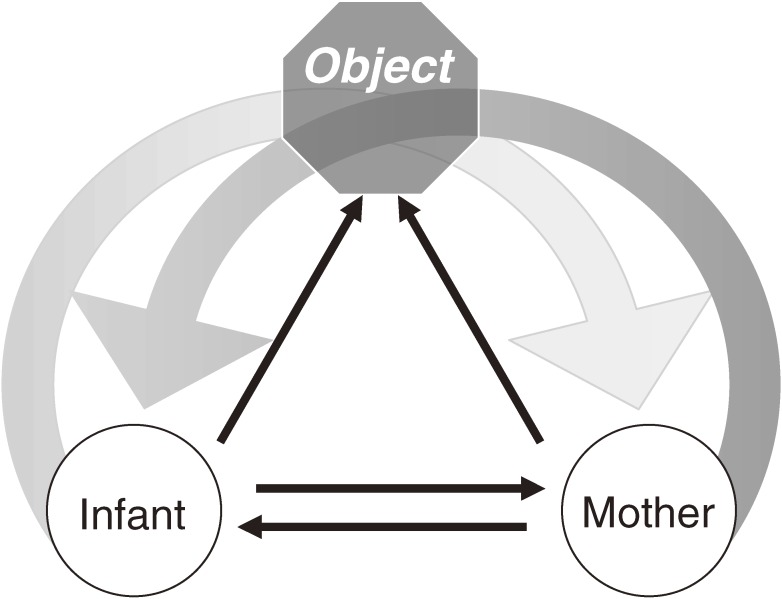
Scheme of dyad and triad Mother-infant intereraction.

In this section, we examined mother-infant interaction using the observational method mentioned above, and propose a development trend/model according to the findings of our analysis. There are many possible aspects to discussing the early mother-infant interaction and communication. In the first stage in analyzing the mother-infant social interaction, we illuminated the transition from face-to-face dyadic format to triadic exchanges with others. The term triadic stands for the three-way transaction between the infant, another person, and objects in the environment. The most widely studied triadic exchange is joint attention, when both the child and mother attend simultaneously to the same object.^7^ Figure 2 shows the most fundamental schema of triadic exchange.

Recent studies have indicated that joint attention has an important role for developing the sociability of children and is also related to developmental disorder like autism.^8^

Joint attention impairments were related to autistic disorders and the absence of early mother infant dyadic behavior.^9^ Additionally, the components of the interactions such as gaze, touch and affection expression are useful variables for understanding the social interaction of an infant.^10^^,^^11^

In this report, we focused on the mother-infant interaction from the perspective of joint attention in early mother and infant interaction.

## METHOD

### Participants

82 mother-and-infant (41boys:41girls) dyad at the Mie JCS group were analyzed. Mean post-conception days were 276 (SD, 9.5) for boy and 276 (SD, 11.1) for girls. Mean birth weight and height for boys and girls was 3106 g (SD, 415.6), 2917 g (SD, 342.3) and 50.2 cm (SD, 1.6), 49.4 (SD, 1.7). They had no problems during the mothers’ pregnancy nor experienced any major illness. Participants met the requirements of observation time for 4, 9, 18, 30 and 42 month observation.

### Observation setting and coding

Mother-infant pairs behavior were observed at an observation room described above. Mother and infant were positioned to confront/interact. Coding and analyzing methods applied were as follows.

### Coding categories

The following 6 categories were selected for analysis—Infant behaviors: gaze-at-mother, gaze-at-toy and vocalization. Mother behaviors: gaze-at-infant, gaze-at-toy and vocalization. These behavior units are the salient to joint attention and are clearly defined.

### Data analysis

Each category status (on/off) is checked by every 5 seconds, and the relative proportion for whole observation session was calculated.

## RESULTS

Mean and standard divisions of 6 indices, infant-gaze-at-mother, infant-gaze-at-toy, infant-vocalization, mother-gaze-at-infant, mother-gaze-at-toy, mother-vocalization by months are at Table 2. There are no gender effects within categories so data bases are combined.

**Table 2.** Mean proportion for whole observation session and standard division (SD) of 6 behavioral indices at 4, 9, 18, 30, 42 month

month	Infant			Mother		
	
	gaze at Mother	gaze at toy	vocalize	gaze at Infant	gaze at toy	vocalize
					
	Mean (SD)	Mean (SD)	Mean (SD)	Mean (SD)	Mean (SD)	Mean (SD)
4	0.48 (0.252)	0.69 (0.356)	0.13 (0.185)	0.96 (0.085)	0.34 (0.254)	0.90 (0.136)
9	0.33 (0.204)	0.85 (0.277)	0.12 (0.155)	0.94 (0.070)	0.64 (0.271)	0.88 (0.132)
18	0.22 (0.185)	0.68 (0.328)	0.28 (0.209)	0.60 (0.246)	0.64 (0.323)	0.89 (0.133)
30	0.21 (0.171)	0.85 (0.178)	0.53 (0.238)	0.53 (0.210)	0.78 (0.230)	0.90 (0.124)
42	0.28 (0.206)	0.86 (0.161)	0.61 (0.252)	0.60 (0.192)	0.81 (0.192)	0.91 (0.113)

These six behaviors are the important component for triad communication. Developmental trends of each category are salient to ordinary development patterns besides infant-gaze-at-mother and mother-gaze-at-infant with vocalization. Infant-gaze-at-toy did not rise steadily and the mother-vocalization increased its occurrence.

In order to search for sources of mother-infant interaction in gaze direction and vocalization, both a correlation analysis and a cluster analysis were conducted. Correlation patterns show the occurrence of the event changes the interaction pattern from toy-to-toy infant to eye-to-eye and toy-to-toy interaction as in a triad pattern (Table 3, 4, 5, 6, 7).

**Table 3.** Correlations between mother infant behavior categories at 4 month

			Infant			Mother		
				
			g at M	g at toy	vo	g at I	g at toy	vo
Infant	gaze at Mother	*Pearson’s r*						
		*P value*						
	gaze at toy	*r*	−0.606					
		*P*	<0.001					
	vocalization	*r*	0.332	−0.389				
		*P*	0.002	<0.001				
Mother	gaze at Infant	*r*	0.068	0.088	0.069			
		*P*	0.547	0.432	0.538			
	gaze at toy	*r*	−0.273	0.589	−0.400	−0.233		
		*P*	0.013	<0.001	<0.001	0.035		
	vocalization	*r*	0.187	0.047	0.145	0.146	0.125	
		*P*	0.093	0.676	0.194	0.191	0.262	

□ = Significant correlation related to the dyad and triad scheme of Mother-Infant interaction (*r* > 0, *P* < 0.01).

**Table 4.** Correlations between mother infant behavior categories at 9 month

			Infant			Mother		
				
			g at M	g at toy	vo	g at I	g at toy	vo
Infant	gaze at Mother	*Pearson’s r*						
		*P value*						
	gaze at toy	*r*	−0.475					
		*P*	<0.001					
	vocalization	*r*	0.062	−0.089				
		*P*	0.583	0.428				
Mother	gaze at Infant	*r*	0.319	−0.226	0.160			
		*P*	0.004	0.041	0.150			
	gaze at toy	*r*	−0.518	0.770	−0.122	−0.407		
		*P*	<0.001	<0.001	0.276	<0.001		
	vocalization	*r*	0.081	−0.028	0.074	0.169	0.084	
		*P*	0.469	0.806	0.509	0.129	0.451	

□ = Significant correlation related to the dyad and triad scheme of Mother-Infant interaction (*r* > 0, *P* < 0.01).

**Table 5.** Correlations between mother infant behavior categories at 18 month

			Infant			Mother		
				
			g at M	g at toy	vo	g at I	g at toy	vo
Infant	gaze at Mother	*Pearson’s r*						
		*P value*						
	gaze at toy	*r*	−0.442					
		*P*	<0.001					
	vocalization	*r*	0.352	−0.110				
		*P*	<0.001	0.325				
Mother	gaze at Infant	*r*	0.588	−0.429	0.091			
		*P*	<0.001	<0.001	0.414			
	gaze at toy	*r*	−0.442	0.895	−0.031	−0.462		
		*P*	<0.001	<0.001	0.780	<0.001		
	vocalization	*r*	0.252	−0.239	0.159	0.220	−0.182	
		*P*	0.023	0.031	0.155	0.047	0.101	

□ = Significant correlation related to the dyad and triad scheme of Mother-Infant interaction (*r* > 0, *P* < 0.01).

**Table 6.** Correlations between mother infant behavior categories at 30 month

			Infant			Mother		
				
			g at M	g at toy	vo	g at I	g at toy	vo
Infant	gaze at Mother	*Pearson’s r*						
		*P value*						
	gaze at toy	*r*	−0.246					
		*P*	0.026					
	vocalization	*r*	0.231	−0.137				
		*P*	0.037	0.219				
Mother	gaze at Infant	*r*	0.431	−0.365	0.144			
		*P*	<0.001	0.001	0.198			
	gaze at toy	*r*	−0.135	0.607	−0.009	−0.321		
		*P*	0.226	<0.001	0.939	0.003		
	vocalization	*r*	0.145	−0.030	0.270	0.000	−0.020	
		*P*	0.195	0.790	0.014	0.998	0.860	

□ = Significant correlation related to the dyad and triad scheme of Mother-Infant interaction (*r* > 0, *P* < 0.01).

**Table 7.** Correlations between mother infant behavior categories at 42 month

			Infant			Mother		
				
			g at M	g at toy	vo	g at I	g at toy	vo
Infant	gaze at Mother	*Pearson’s r*						
		*P value*						
	gaze at toy	*r*	−0.055					
		*P*	0.621					
	vocalization	*r*	0.166	−0.160				
		*P*	0.136	0.150				
Mother	gaze at Infant	*r*	0.348	−0.235	0.082			
		*P*	0.001	0.034	0.465			
	gaze at toy	*r*	−0.080	0.646	0.044	−0.377		
		*P*	0.472	<0.001	0.696	<0.001		
	vocalization	*r*	0.166	−0.145	0.516	0.043	0.131	
		*P*	0.135	0.195	<0.001	0.699	0.242	

□ = Significant correlation related to the dyad and triad scheme of Mother-Infant interaction with vocal channel (*r* > 0, *P* < 0.01).

**Figure 3.**
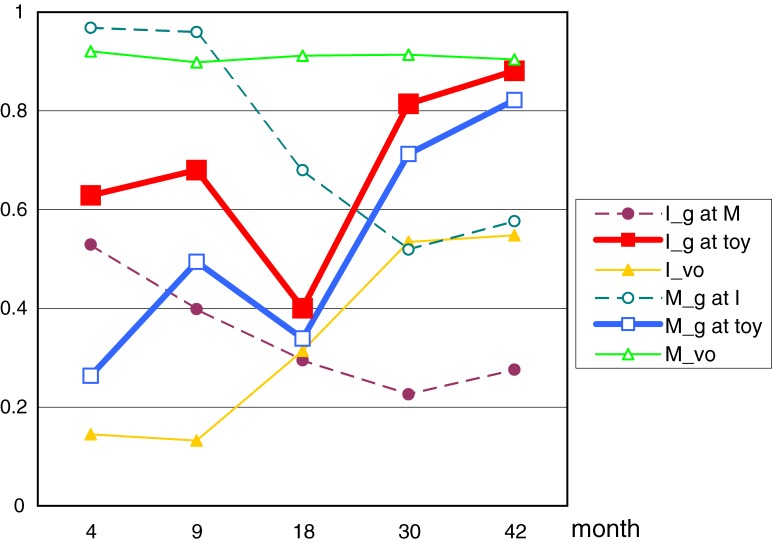
Mean proportion of Cluster1’s behavioral indices at 4, 9, 18, 30, 42 month.

**Figure 4.**
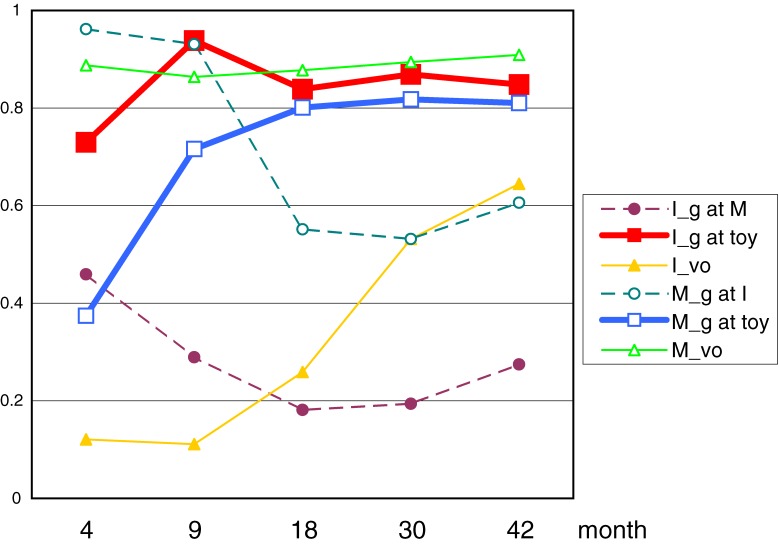
Mean proportion of Cluster2’s behavioral indices at 4, 9, 18, 30, 42 month.

In order to find patterns of mother-infant interaction through the five time-periods, an Hierarchical Cluster Analysis (Ward Method) was conducted. Six behavioral indices for each month (30 variables) were investigated. Two clusters were detected. The result of each cluster analysis’ mean proportions of behavioral categories were calculated for cluster 1 and 2 (Figure 3 and 4). These figures demonstrated the emergence of the month that mother (□) and baby (■) behavior on “Gazing at Toy” rising at c.9month and c.30months respectively.

Cluster1 included 29 infants (boy 13, girl 16), and 13 infants had no siblings, 12 infants had 1, 4 infants had 2. Mean of Birth weight was 2963.9 (g); Birth height 49.6 (cm); pregnancy period 543.4 (day). Cluster2 included 53 infants (boy 28, girl 25), and 30 infants had no siblings, 17 infants had 1, 6 infants had 2. Mean of Birth weight was 3037.3 (g); Birth height 49.9 (cm); pregnancy period 552.9 (day). There were no significant differences on those conditions between Cluster1 and 2.

In order to examine developmental changes between clusters in the percentages of infant-gaze-at-toy, we conducted repeated measure analyses of variance with month (4, 9, 18, 30, and 42 months) as the within-subject factor, and clusters (1, 2) as the between-group factor. LSD tests were used for post hoc analysis. A significant interaction was found between month and cluster (*F*_(3.075, 246.004)_ = 9.907, *P* < 0.001)*, and some simple main effects were found (Cluster1: 9m > 18m < 30m; Cluster2 4m < 9m. 9, 18m: Cluster1 < Cluster2). For more analysis to examine the developmental changes between clusters in the percentages of mother-gaze-at-toy, we conducted repeated measure analyses of variance in the same way. A significant interaction was found between month and cluster (*F*_(3.506, 280.507)_ = 10.449, *P* < 0.001)*, and some simple main effects were found (Cluster1: 4m < 9m > 18m < 30m; Cluster2 4m < 9m. 9, 18, 30m: Cluster1 < Cluster2).

## DISCUSSION

The purposes of this study were to evaluate the observation method and identify the developmental trend of mother-infant interaction from 4 to 42 months of age by observation method. The results indicated that our observation-situation and method-examination categories applied for detecting the mother-infant interaction demonstrate an alternate assessment approach for cohort study.

Reflecting about the pattern of behavioral change in mother-infant interaction, we found two clusters in developmental trends of mother-infant interaction. Mean proportion and the correlation of infant/mother gaze-at-toy, gaze at mother/infant, and vocalization indicated that the mother- and infant-gaze-at-toy correlate from 4 to 42 months, however mother- and infant-vocalization of are correlated after 9 months of age.

This is not to suggest a triad interaction but simply proposes that the gaze-at-toy and gaze-at-each-other phases occur together at around 9 months. This result is salient to the joint attention model recorded at around 9 months.^12^^,^^13^

However this model has not yet been examined in the light of a triad relationship with its inherent changing pattern of development.

Using cluster analysis, we discovered two types of changing related to mother-infant interaction. The first is that the same pattern of ordinary model that the triad relations happened at around 9 month (Figure 4), and the other could be described as a type of “catch up” group which increased after 9 months of age (Figure 3). We refer to this type of infant as a “slow starter”. We have not analyzed the differences between these two groups as yet, but future studies and their resulting analysis will expand further our understandings of the social behavior development of infants.

## Footnotes

*The tests were used modified degrees of freedom by Greenhouse-Geisser’s ε.
